# Thermodynamic parameters of phenylglycine interaction with UO_2_^2+^, La^3+^ and Zr^4+^

**DOI:** 10.1186/s13065-023-00966-7

**Published:** 2023-06-15

**Authors:** Farid I. El-Dossoki, AbdAllah AbdEl-Wahab Mohamed

**Affiliations:** 1 Department of Chemistry, Faculty of Science, Port Said University, Port Said, Egypt; 2 Department of Chemistry, Faculty of Science, Al-Azhar University, Nasr City, Cairo, Egypt

**Keywords:** Thermodynamic, Stability constant, Phenylglycine

## Abstract

Phenylglycine interactions with (UO_2_^2+^, La^3+^ and Zr^4+^) transition metal ions were studied at different ionic strengths and different temperature degrees applying Bjerrum’s method. The work determine and discuss both the thermodynamic stabilities and the degree of interactions $\left(\acute{{\text{n}}}\right)$. Also the work calculate and discuss the thermodynamic parameters of interactions of phenylglycine with (UO_2_^2+^, La^3+^ and Zr^4+^). The variables that govern the interaction between phenylglycine and the metal ions under investigation were related to the nature of the amino acid reactive species as well as to the nature of M^+^ such as the valence and radius of the ion. It was observed that reactions between the M^+^ and L^−^ were the most likely to occur. It was determined that the pH values affect the degree of complex formation $\overline{{\text{n}}}$ as well as the production of various reactive spices. When the range of degree of interaction was more than 0.5 and less than 1.15, forming 1:1 stoichiometric complex. Additionally, it was shown that the stability of the complexes produced between phenylglycine and M^Z+^ increased in the subsequent order, which was in good accord with the Irving–Williams order.

## Introduction

Several authors have been curious about investigating the communication between transitional metal ions and amino acids due to their relevance at the present time, the importance of amino acids as the building blocks of protein molecules and hormones, and the fact that transitional metal ions are present in all plant and animal organisms [1–8]. As a result, this research is a crucial step in the overall framework of our laboratory [9–12], in this study, the complexation mechanisms between phenylglycine and various transition metal ions (UO_2_^2+^, La^3+^ and Zr^4+^) are examined in terms of a unique thermodynamic technique. By using the Bjerrum pH titration technique, the parameters needed for examining these reactions were established at three ionic strengths [(μ = 0.05, 0.10, and 0.15 M) KNO_3_] and three temperatures [(25, 35, and 45) °C] in aqueous solutions [13–15]. In order to perform the Bjerrum pH titration procedure, the ionization constants (pK_011_ and pK_012_) for pure phenylglycine must be determined at identical ionic strength and temperature circumstances. The quantities of pK_011_ and pK_012_ have already been determined by us [9]. The degree of complexation of the investigated systems (${\overline{\text{n}}}$) has been calculated, and by implementing this information, the stoichiometric stability constants (K), the thermodynamic stability constants (K°), the standard thermodynamic parameters (ΔG°, ΔH°, and ΔS°), and standard thermodynamic differences (ΔΔG°, ΔΔH° and ΔΔS°) were computed. Throughout the analysis of these results, the variables that influence the complexity of the processes examined from the perspective of thermodynamics have been identified.

## Experimental

### Materials

All chemicals used in this research (phenylglycine, NaOH, anhydrous La (NO_3_)_3_ (98.50% purity), Zr (NO_3_)_4_ (99.20% purity), and UO_2_ (NO_3_)_2_ (98.90% purity) were supplied from Riedel-deHäen Co. Bidistilled water was used to prepare the solutions after the deionization process. Just before the pH examinations, the solutions were made by dissolving the precise weight of each salt in the bidistilled water. Equivalent parts of pure phenylglycine and sodium hydroxide solution were combined to produce sodium phenylglycinate. The accurate concentrations of transition metal nitrate solutions were calculated by standardization with EDTA [16–18].

### Procedure

#### Apparatus

The readings of pH were taken using an Orion Research pH meter with a glassy electrode. Three buffers, with pH values of 4, 7, and 10, were used to calibrate the pH meter. The next equation was used to find the two-pH meter's correcting coefficients in the acidic and basic ranges, respectively [9, 10, 16, 17]:

1 $$ {\text{pH}} = {7}.00 + \left( {{\text{pH}}_{{\text{meter}}} - { 7}.00} \right){\text{C}}_{{\text{meter}}} $$

#### Calculating phenylglycine's ionization constants

The steps for determining phenylglycine's ionization constants and the results for p*K*_011_ and p*K*_012_ were described in our earlier study [9]. According to the below equilibria, Bjerrum's pH-titration method presupposes the existence of the reactive species [10, 16, 17]:

2 $$ H_2 L^{ + } {\mathop{\Longleftrightarrow}\limits^{K_{011}}} HL + H^{ + } $$

3 $$ HL{\mathop{\Longleftrightarrow}\limits^{K_{012}}} L^{ - } + H^{ + } $$

where H_2_L^+^, HL, and L^−^ refer to the diprotonated phenylglycine, the monoprotonated phenylglycine, and the phenylglycine anion, respectively.

The next equations were applied to determine the quantities of phenylglycine's ionization constants (K_011_ and K_012_) [10, 16, 17]:

4 $$ pK_{011} = - \log_{10} \left[ {H^+ } \right] + \log_{10 } \frac{{ \left[ {Cl^- } \right] + \left[ {OH^- } \right] - \left[ {H^+ } \right]}}{{L_{tot^- } \left\{ {\left[ {Cl^- } \right] - \left[ {OH^- } \right] - \left[ {H^+ } \right]} \right\}}} $$

5 $$ pK_{012} = - \log_{10} \left[ {H^+ } \right] + \log_{10 } \frac{{ L_{tot^- } \left\{ {\left[ {H^+ } \right] + \left[ {Na^+ } \right] - \left[ {OH^- } \right]} \right\}}}{{\left[ {H^+ } \right] + \left[ {Na^+ } \right] - \left[ {OH^- } \right]}} $$

wherein L_tot_ denotes the total concentration of phenylglycine. The concentrations of hydrogen ion [H^+^] and hydroxyl ion [OH^−^] are determined by pH and the water dissociation constant, respectively (where pH =  − log γ_+_[H^+^] and pK_w_ = pH + pOH), and the activity coefficient (γ_+_) was established by the Debye–Huckel limiting law as modified by Robinson and Stokes. Cl^−^ and Na^+^ are from the amount of titrant. The quantities of the phenylglycine ionization constants (*K*_011_ and *K*_012_), which have been published in our earlier study [9], are shown in Table 1.

**Table 1:** The values of pure phenylglycine's ionization constants at various temperatures and ionic strengths (μ) [9]

T °C	μ					
	p*K*_011_			p*K*_012_		
	0.05	0.10	0.15	0.05	0.10	0.15
25	4.21	4.11	4.05	9.38	9.29	9.23
30	4.18	4.07	3.99	9.33	9.25	9.20
35	4.15	4.03	3.94	9.28	9.22	9.17
40	4.10	3.98	3.90	9.27	9.21	9.16
45	4.05	3.94	3.86	9.26	9.20	9.15

#### Estimation of the stability constants

As described in our earlier study, the method for determining the stability constants of phenylglycine complexes with UO_2_^2+^, La^3+^ and Zr^4+^ was used [9].

The common equation below can be used to obtain the overall stability constant *K* [9, 12–14, 16, 17]:

6 $$ \frac{{{\overline{\text{n}}}}}{{\left( {1 - {\overline{\text{n}}}} \right)\left[ {\text{L}} \right]}} = \mathop{\sum}\limits_{i = 1}^j \frac{{\left( {i - {\overline{\text{n}}}} \right)}}{{\left( {1 - {\overline{\text{n}}}} \right)}}K_i \left[ {\text{L}} \right]^{i - 1} $$

where *j* is the greatest number of ligands that may be attached to the metal ion, *i* is a numerical value dependent on the kind of the complex produced (*i* may be 1, 2, or 3 according to L:M stoichiometric complex, [L] refers to the concentration of unbonded ligand, and ${\overline{\text{n}}}$ is the degree of complex formation calculated from the following equation [9, 13, 14, 16, 17]:

7 $$ {\overline{\text{n}}} = \frac{{\text{Bound ligand}}}{{\text{Total metal ion concentration}}} = \frac{{{\text{L}}_{{\text{bound}}} }}{{{\text{C}}_{\text{M}} }} = \frac{{{\text{L}}_{{\text{total}}} - {\text{L}}_{{\text{free}}} }}{{{\text{C}}_{\text{M}} }} $$

During the titration process, L_total_ was determined by executing the dilution law soon after every addition of the ligand, and L_free_ equal to the sum of [H_2_L^+^] + [HL] + [L^−^]. Previous studies [20, 21] provided more information on how to calculate the [L] and ${\overline{\text{n}}}$ values. The estimated values of $\overline{{\text{n}}}$ are in the range (0.0 < ${\overline{\text{n}}}$ ≤ 1.05) indicate the formation of a 1:1 stoichiometric complex. Applying Eq. 6 in the proposed scheme reaction I, when *i* = 1 for the formed 1:1 stoichiometric complex (ML), the equation will be changed to the subsequent form:

8 $$ \frac{{{\overline{\text{n}}}}}{{\left( {1 - {\overline{\text{n}}}} \right)\left[ {\text{L}} \right]}} = K_{110} $$

When *i* = 2 in the ML_2_ case, the proposed equation changed to e next equation:

9 $$ \frac{{{\overline{\text{n}}}}}{{\left( {1 - {\overline{\text{n}}}} \right)\left[ {\text{L}} \right]}} = K_{120} \frac{{\left( {2 - {\overline{\text{n}}}} \right)}}{{\left( {1 - {\overline{\text{n}}}} \right)}}\left[ {\text{L}} \right] $$

Consequently, the following is a form of the reaction scheme (I) overall equation:

10 $$ \frac{{{\overline{\text{n}}}}}{{\left( {1 - {\overline{\text{n}}}} \right)\left[ {\text{L}} \right]}} = K_{110} + K_{120} \frac{{\left( {2 - {\overline{\text{n}}}} \right)}}{{\left( {1 - {\overline{\text{n}}}} \right)}}\left[ {\text{L}} \right] $$

A plot of $\frac{{{\overline{\text{n}}}}}{{\left( {1 - {\overline{\text{n}}}} \right)\left[ {\text{L}} \right]}}$ against $\frac{{\left( {2 - {\overline{\text{n}}}} \right)}}{{\left( {1 - {\overline{\text{n}}}} \right)}}\left[ {\text{L}} \right]$, has a slope of *K*_120_ and intercept of *K*_110_.

In the same way, *K*_111_ and *K*_122_ (for the recommended reaction II) may be determined from the intercept and slope values according to the next equation:

11 $$ \frac{{{\overline{\text{n}}}\left[ {{\text{H}}^+ } \right]}}{{\left( {1 - {\overline{\text{n}}}} \right)\left[ {{\text{HL}}} \right]}} = K_{111} + K_{122} \frac{{\left( {2 - {\overline{\text{n}}}} \right)\left[ {{\text{HL}}} \right]}}{{\left( {1 - {\overline{\text{n}}}} \right)\left[ {{\text{H}}^+ } \right]}} $$

Utilizing the Davies equation, the thermodynamic stability constant K° was determined at each temperature and ionic strength:

12 $$ \log K = \log K^{\circ} - \frac{0.5097Z_+ Z_- \sqrt \mu }{{\left( {1 + 1.5\sqrt \mu } \right)}} - \, 0.{3}\mu $$

## Calculations

### Ionization constants of phenylglycine

In our earlier study [9], we reported the ionization constants of phenylglycine (*K*_011_ and *K*_012_).

### Calculation of the stability constants of phenylglycine-MZ^+^ complexes

According to earlier publications [9, 10, 12–14, 16, 17], the stability constants of phenylglycine complexes with UO_2_^2+^, La^3+^ and Zr^4+^ and the proposed mechanism of association were calculated.

The concentrations of H_2_L^+^, HL and L^−^ that exist in the reaction solution were calculated using *K*_011_ and *K*_012_ for pure phenylglycine. As a result, the indicated mechanisms were used to carry out the overall reaction that was considered to be most likely [9]. The overall stability constants of the complexes produced by the proposed reaction (I) are *K*_110_ and *K*_120_, while those of the complexes produced by the proposed reaction (II) are *K*_111_ and *K*_122_ [18–21].

#### Reaction Scheme (I)

To keep things simple, let's limit our discussion to the complexation of divalent ions (z = 2). According to the following, the deprotonated phenylglycine anion L^−^ can function as an interacting ligating species:

13 $$  {\text{M}}^{{2 + }}  + {\text{L}}^{ - } \overset {K_{{110}} } \longleftrightarrow {\text{ML}}^{{\text{ + }}} \quad K_{{110}}  = \frac{{[{\text{ML}}^{{{ + }}} }}{{[{\text{M}}^{{2 + }} ][{\text{L}}^{ - } ]}} $$

14 $$ {\text{M}}^{{2 + { }}} + 2{\text{L}}^{ - } \overset {K_{120} } \longleftrightarrow {\text{ML}}_{{2{ }}} \quad K_{120} = \frac{{\left[ {{\text{ML}}_{2} } \right]}}{{\left[ {{\text{M}}^{2 + } } \right]\left[ {{\text{L}}^{ - } } \right]^{2} }} $$

where *K*_110_ and *K*_120_ are the complexes' overall stability constants after undergoing the proposed reaction (I).

#### Reaction Scheme (II)

Based on the following equilibrium, phenylglycine could function as a ligating gene with proton release:

15 $$ {\text{M}}^{2 +} + {\text{HL }}\mathop{\longleftrightarrow}\limits^{K_{111} } {\text{ML}}^+ + {\text{H}}^+ \quad K_{111} = \frac{{\left[ {{\text{ML}}^+ } \right]\left[ {{\text{H}}^+ } \right]}}{{\left[ {{\text{M}}^{2 + } } \right]\left[ {{\text{HL}}} \right]}} $$

16 $$ {\text{M}}^{2 +} + 2{\text{HL}}\mathop{\longleftrightarrow}\limits^{K_{122} } {\text{ML}}_2 + { }2{\text{H}}^+ \quad K_{122} = \frac{{\left[ {{\text{ML}}_2 } \right]\left[ {{\text{H}}^+ } \right]^2 }}{{\left[ {{\text{M}}^{2 + } } \right]\left[ {{\text{HL}}} \right]^2 }} $$

where *K*_111_ and *K*_122_ are the complexes' overall stability constants after undergoing the proposed reaction (II).

The production of a 1:1 stoichiometric compound is suggested by the measured quantities of ${\overline{\text{n}}}$ being in the range (from 0.0 to 1.15). The thermodynamic functions were calculated using the equations:

17a $$ \Delta G^{\circ} = - RT \, ln \, K = \Delta H^{\circ} - T\Delta S^{\circ} $$

17b $$ \Delta S^{\circ} = \frac{{\Delta H^{\circ} - \Delta G^{\circ} }}{T} $$

where ΔG° is the standard free energy change, ΔH° the standard enthalpy change, and ΔS° the standard entropy change. It is possible to calculate ΔH° value by using temperature dependence method [9–12]. A plot of Log K° vs the reciprocal of absolute temperature (1/T) gives a straight line with a slope (obtained from least squares analysis) equal to − ΔH°/2.303R. Then, ΔS° can be calculated by Eq. (17b). In this study, every computation was pc-programmed.

## Results and discussion

The degree of complex formation (${\overline{\text{n}}}$) values are gathered in Tables 2, 3 and 4.

**Table 2:** Summary of ${\overline{\text{n}}}$ data for the UO_2_^2+^ complexes with phenylglycine at different ionic strengths and different temperatures

Temp. °C	μ	pH	${\overline{\text{n}}}$	[H_2_L^+^]	[HL]	[L^−^]
25	0.05	4.45	0.07	2.76 × 10^–4^	3.90 × 10^–4^	3.76 × 10^–9^
		4.90	0.43	1.61 × 10^–4^	6.39 × 10^–4^	1.74 × 10^–8^
		5.05	0.64	1.26 × 10^–4^	7.08 × 10^–4^	2.72 × 10^–8^
		5.27	0.84	8.48 × 10^–5^	7.90 × 10^–4^	5.04 × 10^–8^
		5.49	1.06	5.48 × 10^–5^	8.48 × 10^–4^	8.97 × 10^–8^
	0.10	4.41	0.07	2.60 × 10^–4^	4.08 × 10^–4^	4.14 × 10^–9^
		4.90	0.41	1.82 × 10^–4^	6.73 × 10^–4^	2.13 × 10^–8^
		5.07	0.61	1.46 × 10^–4^	7.49 × 10^–4^	3.49 × 10^–8^
		5.26	0.83	7.37 × 10^–5^	8.03 × 10^–4^	5.89 × 10^–8^
		5.37	1.03	2.78 × 10^–5^	8.57 × 10^–4^	1.02 × 10^–7^
	0.15	4.38	0.07	2.57 × 10^–4^	4.17 × 10^–4^	4.47 × 10^–9^
		4.91	0.40	1.28 × 10^–4^	7.04 × 10^–4^	2.56 × 10^–8^
		5.10	0.61	9.13 × 10^–5^	7.78 × 10^–4^	4.38 × 10^–8^
		5.26	0.83	6.70 × 10^–5^	8.25 × 10^–4^	6.71 × 10^–8^
		5.45	1.05	4.54 × 10^–5^	8.66 × 10^–4^	1.09 × 10^–7^
35	0.05	4.35	0.09	2.81 × 10^–4^	3.68 × 10^–4^	3.55 × 10^–9^
		4.78	0.45	1.73 × 10^–4^	6.09 × 10^–4^	1.58 × 10^–8^
		4.94	0.65	1.35 × 10^–4^	6.87 × 10^–4^	2.58 × 10^–8^
		5.14	0.86	9.51 × 10^–5^	7.67 × 10^–4^	4.57 × 10^–8^
		5.35	1.07	6.34 × 10^–5^	8.29 × 10^–4^	8.01 × 10^–8^
	0.10	4.30	0.09	2.66 × 10^–4^	3.89 × 10^–4^	3.66 × 10^–9^
		4.75	0.43	1.56 × 10^–4^	6.41 × 10^–4^	1.70 × 10^–8^
		4.93	0.64	1.16 × 10^–4^	7.23 × 10^–4^	2.91 × 10^–8^
		5.10	0.85	8.53 × 10^–5^	7.85 × 10^–4^	4.67 × 10^–8^
		5.32	1.06	5.53 × 10^–5^	8.45 × 10^–4^	8.34 × 10^–8^
	0.15	4.26	0.06	2.36 × 10^–4^	4.41 × 10^–4^	4.31 × 10^–9^
		4.75	0.40	1.22 × 10^–4^	7.07 × 10^–4^	2.14 × 10^–8^
		4.94	0.61	8.73 × 10^–5^	7.81 × 10^–4^	3.65 × 10^–8^
		5.08	0.83	6.66 × 10^–5^	8.22 × 10^–4^	5.31 × 10^–8^
		5.28	1.05	4.43 × 10^–5^	8.66 × 10^–4^	8.86 × 10^–8^
45	0.05	4.23	0.11	2.81 × 10^–4^	3.52 × 10^–4^	2.66 × 10^–9^
		4.64	0.47	1.82 × 10^–4^	5.85 × 10^–4^	1.14 × 10^–8^
		4.81	0.66	1.41 × 10^–4^	6.71 × 10^–4^	1.93 × 10^–8^
		4.99	0.87	1.04 × 10^–4^	7.47 × 10^–4^	3.25 × 10^–8^
		5.20	1.08	6.97 × 10^–5^	8.15 × 10^–4^	5.75 × 10^–8^
	0.10	4.18	0.11	2.68 × 10^–4^	3.65 × 10^–4^	3.63 × 10^–9^
		4.61	0.45	1.67 × 10^–4^	6.11 × 10^–4^	1.19 × 10^–8^
		4.79	0.65	1.26 × 10^–4^	6.98 × 10^–4^	2.05 × 10^–8^
		4.95	0.86	9.51 × 10^–5^	7.62 × 10^–4^	3.24 × 10^–8^
		5.15	1.08	6.48 × 10^–5^	8.23 × 10^–4^	5.54 × 10^–8^
	0.15	4.12	0.12	2.63 × 10^–4^	3.57 × 10^–4^	2.53 × 10^–9^
		4.60	0.44	1.55 × 10^–4^	6.33 × 10^–4^	1.35 × 10^–8^
		4.78	0.64	1.16 × 10^–4^	7.17 × 10^–4^	2.32 × 10^–8^
		4.93	0.86	8.85 × 10^–5^	7.74 × 10^–4^	3.54 × 10^–8^
		5.09	1.08	6.50 × 10^–5^	8.21 × 10^–4^	5.42 × 10^–8^

**Table 3:** Summary of $\overline{{\text{n}}}$ data for La^3+^ complexes with phenylglycine at different ionic strengths and different temperatures

Temp. °C	μ	pH	${\overline{\text{n}}}$	[H_2_L^+^]	[HL]	[L^−^]
25	0.05	4.60	0.02	2.39 × 10^–4^	4.76 × 10^–4^	6.49 × 10^–9^
		8.09	0.14	6.06 × 10^–8^	9.13 × 10^–4^	9.11 × 10^–5^
		9.75	0.42	3.52 × 10^–9^	1.12 × 10^–3^	1.93 × 10^–3^
		9.79	0.52	1.67 × 10^–9^	1.01 × 10^–3^	2.07 × 10^–3^
		10.25	0.55	1.75 × 10^–9^	1.06 × 10^–3^	2.17 × 10^–3^
	0.10	6.00	0.11	4.76 × 10^–8^	7.16 × 10^–4^	3.57 × 10^–8^
		8.40	0.15	6.49 × 10^–8^	9.78 × 10^–4^	9.76 × 10^–5^
		9.69	0.41	3.47 × 10^–9^	9.99 × 10^–4^	1.90 × 10^–3^
		9.73	0.51	3.24 × 10^–9^	9.98 × 10^–4^	2.05 × 10^–3^
		10.22	0.54	3.41 × 10^–9^	1.05 × 10^–3^	2.16 × 10^–3^
	0.15	7.30	0.03	3.27 × 10^–8^	7.36 × 10^–4^	9.74 × 10^–9^
		8.65	0.04	3.25 × 10^–8^	9.82 × 10^–4^	1.96 × 10^–4^
		9.63	0.41	3.44 × 10^–9^	9.92 × 10^–4^	1.89 × 10^–3^
		9.66	0.51	3.21 × 10^–9^	9.91 × 10^–4^	2.03 × 10^–3^
		10.14	0.53	3.37 × 10^–9^	1.04 × 10^–3^	2.13 × 10^–3^
35	0.05	4.48	0.05	2.51 × 10^–4^	4.43 × 10^–4^	5.77 × 10^–9^
		8.05	0.49	1.48 × 10^–7^	9.73 × 10^–4^	4.71 × 10^–5^
		8.51	0.60	5.15 × 10^–8^	9.74 × 10^–4^	1.36 × 10^–4^
		9.09	0.96	1.46 × 10^–8^	9.82 × 10^–4^	4.86 × 10^–4^
		9.15	1.08	1.19 × 10^–8^	9.84 × 10^–4^	6.00 × 10^–4^
	0.10	7.76	0.22	2.32 × 10^–7^	9.77 × 10^–4^	2.66 × 10^–5^
		8.26	0.41	7.33 × 10^–8^	9.76 × 10^–4^	8.39 × 10^–5^
		8.60	0.55	3.35 × 10^–8^	9.77 × 10^–4^	1.84 × 10^–4^
		9.16	1.00	9.33 × 10^–9^	9.87 × 10^–4^	6.74 × 10^–4^
		9.23	1.11	7.96 × 10^–9^	9.89 × 10^–4^	7.94 × 10^–4^
	0.15	8.00	0.19	9.53 × 10^–8^	9.78 × 10^–4^	5.25 × 10^–5^
		8.69	0.47	1.95 × 10^–8^	9.80 × 10^–4^	2.58 × 10^–4^
		8.87	0.57	1.29 × 10^–8^	9.82 × 10^–4^	3.91 × 10^–4^
		9.23	0.99	5.68 × 10^–9^	9.91 × 10^–4^	9.04 × 10^–4^
		9.28	1.11	5.07 × 10^–9^	9.92 × 10^–4^	1.02 × 10^–3^
45	0.05	4.33	0.07	2.60 × 10^–4^	4.09 × 10^–4^	3.90 × 10^–9^
		7.34	0.49	6.01 × 10^–7^	9.71 × 10^–4^	9.46 × 10^–5^
		7.90	0.71	1.66 × 10^–7^	9.70 × 10^–4^	3.43 × 10^–4^
		8.25	0.91	7.39 × 10^–8^	9.70 × 10^–4^	7.68 × 10^–4^
		8.46	1.11	4.56 × 10^–8^	9.71 × 10^–4^	1.25 × 10^–3^
	0.10	6.50	0.02	3.42 × 10^–6^	9.73 × 10^–4^	1.47 × 10^–6^
		7.64	0.48	2.48 × 10^–7^	9.73 × 10^–4^	2.02 × 10^–5^
		8.10	0.68	8.59 × 10^–8^	9.73 × 10^–4^	5.84 × 10^–5^
		8.39	0.87	4.41 × 10^–8^	9.74 × 10^–4^	1.14 × 10^–4^
		8.57	1.05	2.92 × 10^–8^	9.76 × 10^–4^	1.73 × 10^–4^
	0.15	7.39	0.24	3.87 × 10^–7^	9.76 × 10^–4^	1.29 × 10^–5^
		7.92	0.45	1.14 × 10^–7^	9.75 × 10^–4^	4.36 × 10^–5^
		8.32	0.63	4.56 × 10^–8^	9.77 × 10^–4^	1.10 × 10^–4^
		8.66	0.97	2.09 × 10^–8^	9.81 × 10^–4^	2.41 × 10^–4^
		8.76	1.15	1.66 × 10^–8^	9.83 × 10^–4^	3.04 × 10^–4^

**Table 4:** Summary of ${\overline{\text{n}}}$ data for Zr^4+^ complexes with phenylglycine at different ionic strengths and different temperatures

Temp. °C	μ	pH	${\overline{\text{n}}}$	[H_2_L^+^]	[HL]	[L^−^]
25	0.05	3.34	0.02	2.07 × 10^–4^	2.27 × 10^–5^	1.70 × 10^–11^
		3.50	0.42	2.78 × 10^–4^	4.40 × 10^–5^	4.76 × 10^–11^
		3.64	0.61	3.16 × 10^–4^	6.90 × 10^–5^	1.03 × 10^–10^
		4.05	0.96	7.37 × 10^–5^	1.89 × 10^–4^	7.26 × 10^–10^
		4.39	1.10	2.84 × 10^–5^	3.49 × 10^–4^	2.93 × 10^–9^
	0.10	3.51	0.10	2.69 × 10^–4^	5.27 × 10^–5^	1.37 × 10^–11^
		3.63	0.36	3.03 × 10^–4^	7.83 × 10^–5^	1.32 × 10^–10^
		3.77	0.55	2.42 × 10^–4^	1.15 × 10^–4^	2.18 × 10^–9^
		4.80	0.91	7.62 × 10^–5^	6.25 × 10^–4^	1.55 × 10^–8^
		5.45	1.05	3.29 × 10^–5^	8.56 × 10^–4^	9.58 × 10^–8^
	0.15	3.60	0.13	2.89 × 10^–4^	7.78 × 10^–5^	1.39 × 10^–10^
		3.92	0.49	3.20 × 10^–4^	1.80 × 10^–4^	6.70 × 10^–10^
		4.21	0.63	2.89 × 10^–4^	3.17 × 10^–4^	2.30 × 10^–9^
		5.19	0.84	7.67 × 10^–5^	8.04 × 10^–4^	5.57 × 10^–8^
		5.77	1.03	2.29 × 10^–5^	9.14 × 10^–4^	2.41 × 10^–7^
35	0.05	3.32	0.03	1.94 × 10^–4^	2.37 × 10^–5^	2.13 × 10^–11^
		3.52	0.41	2.81 × 10^–4^	5.44 × 10^–5^	7.77 × 10^–11^
		3.66	0.59	3.15 × 10^–4^	8.41 × 10^–5^	1.66 × 10^–10^
		4.08	0.94	3.22 × 10^–4^	2.26 × 10^–4^	1.17 × 10^–9^
		4.41	1.07	3.62 × 10^–4^	3.94 × 10^–4^	4.37 × 10^–9^
	0.10	3.35	0.02	1.94 × 10^–4^	3.18 × 10^–5^	3.36 × 10^–11^
		3.57	0.39	2.81 × 10^–4^	7.64 × 10^–5^	1.34 × 10^–10^
		3.79	0.60	2.95 × 10^–4^	2.44 × 10^–5^	1.95 × 10^–10^
		4.69	0.95	1.68 × 10^–4^	6.03 × 10^–4^	1.40 × 10^–8^
		5.18	1.09	7.29 × 10^–5^	8.07 × 10^–4^	5.78 × 10^–8^
	0.15	3.52	0.16	2.51 × 10^–4^	8.54 × 10^–5^	1.52 × 10^–10^
		3.80	0.42	2.42 × 10^–4^	2.88 × 10^–4^	1.34 × 10^–10^
		4.70	0.56	2.04 × 10^–4^	4.46 × 10^–4^	4.94 × 10^–10^
		5.20	0.83	5.24 × 10^–5^	8.53 × 10^–4^	7.29 × 10^–8^
		5.30	1.05	4.25 × 10^–5^	8.70 × 10^–4^	9.32 × 10^–7^
45	0.05	3.30	0.04	1.79 × 10^–4^	2.63 × 10^–5^	2.34 × 10^–11^
		3.53	0.40	2.78 × 10^–4^	6.95 × 10^–5^	1.05 × 10^–10^
		3.68	0.58	3.08 × 10^–4^	1.09 × 10^–4^	2.32 × 10^–10^
		4.11	0.90	2.97 × 10^–4^	2.82 × 10^–4^	1.62 × 10^–9^
		4.44	1.03	2.29 × 10^–4^	4.65 × 10^–4^	5.70 × 10^–9^
	0.10	3.31	0.05	1.67 × 10^–4^	3.06 × 10^–5^	2.98 × 10^–11^
		3.52	0.41	2.59 × 10^–4^	7.72 × 10^–5^	1.22 × 10^–10^
		3.68	0.57	2.93 × 10^–4^	1.26 × 10^–4^	2.87 × 10^–10^
		4.10	0.89	2.77 × 10^–4^	3.14 × 10^–4^	1.88 × 10^–9^
		4.41	1.02	2.13 × 10^–4^	4.91 × 10^–4^	6.02 × 10^–9^
	0.15	3.32	0.05	1.61 × 10^–4^	3.46 × 10^–5^	1.89 × 10^–11^
		3.53	0.41	2.52 × 10^–4^	8.77 × 10^–5^	1.60 × 10^–10^
		3.66	0.58	2.79 × 10^–4^	1.31 × 10^–4^	3.21 × 10^–10^
		4.07	0.89	2.67 × 10^–5^	3.23 × 10^–4^	2.04 × 10^–9^
		4.37	1.01	2.06 × 10^–5^	4.97 × 10^–4^	6.25 × 10^–9^

Potentiometric pH studies, however, can only give the degree of formation of the system (or ligand number) $\overline{{\text{n}}}$, but cannot ascertain the mode of bonding. From the collected data in Tables 2, 3 and 4, it is obvious that the values of the degrees of complexation are directly proportional to the pH readings, and this increase in the values of (${\overline{\text{n}}}$) is attributed to the continuous increase in the concentration of sodium phenylglycinate during the titration process. By choosing one of the temperatures, let it be at 25 °C, and one of the ionic strengths, let it be 0.05 M for example, we detected that at low pH range (4.45 ≤ pH ≤ 4.90 for UO_2_^2+^ and 3.34 ≤ pH ≤ 3.50 for Zr^4+^ complexes), H_2_L^+^ has very little interaction, as evidenced by the extremely low ${\overline{\text{n}}}$ values (less than 0.5). At pH values (4.90 < pH ≤ 5.27 for UO_2_^2+^, 9.75 < pH ≤ 10.25 for La^3+^ and 3.50 < pH ≤ 4.39 for Zr^4+^), the concentration of HL is large and appears to be steady, whereas the concentrations of L^−^ and H_2_L^+^ changed. We see that in this pH range, for 0.5 < $\overline{{\text{n }}}$ ≤ 1.10 (i.e., 1:1 complex was produced).

It is noteworthy to note the effect of the endothermic nature of complexation processes between La^3+^ and phenylglycine on the values of (${\overline{\text{n}}}$) clearly, where (${\overline{\text{n}}}$) values decrease with a decrease in temperature (at 25 °C) and increase with a gradual increase in temperature (at 35 °C and 45 °C). This behavior is attributed to the high positive value of enthalpy change, where ΔH° = 128.04 kJ mol^−1^ and 139.00 kJ mol^−1^ for scheme (I) and scheme (II) respectively (see Table 7).

The titrations were terminated because complex formation is seen as pH rises and insoluble precipitates are produced. Complexes have no possibility of forming at high pH levels because the hydroxide precipitates. Tables 5 and 6 list the stoichiometric and thermodynamic stability constants.

**Table 5:** Stability constants of M^Z+^-phenylglycine complexes according to the reaction scheme I and scheme II

Metal ion	μ	log K_110_ (Scheme I)			log K_111_ (Scheme II)		
		25 °C	35 °C	45 °C	25 °C	35 °C	45 °C
UO_2_^2+^	0.05	7.13	7.52	7.75	− 2.03	− 1.76	− 1.52
	0.10	7.23	7.48	7.71	− 2.06	− 1.74	− 1.50
	0.15	7.22	7.31	7.70	− 2.01	− 1.84	− 1.45
La^3+^	0.05	3.44	4.28	5.00	− 5.94	− 5.00	− 4.27
	0.10	2.92	3.94	4.70	− 6.37	− 5.27	− 4.51
	0.15	2.47	3.48	4.37	− 6.76	− 5.66	− 4.78
Zr^4+^	0.05	10.14	9.95	9.77	0.76	0.67	0.51
	0.10	9.67	9.75	9.73	0.38	0.43	0.52
	0.15	9.74	9.68	9.62	− 0.09	− 0.06	0.48

**Table 6:** Thermodynamic stability constants of M^Z+^-phenylglycine complexes

Metal ion	Log K°_110_ (Scheme I)			Log K°_111_ (Scheme II)		
	25 °C	35 °C	45 °C	25 °C	35 °C	45 °C
UO_2_^2+^	7.10	7.65	7.78	− 2.05	− 1.70	− 1.56
La^3+^	3.91	4.70	5.52	− 5.54	− 4.65	− 4.01
Zr^4+^	10.25	10.06	9.86	1.08	1.00	0.53

Uncertainty of Log K°_110_ and Log K°_111_, is 0.01

From Tables 5 and 6, we can observe that logK_110_ >  >  > logK_111_. To understand the distinctions between the two proposed reactions of the metal ion with L^−^ and HL, the following equations were proposed:

18 $$  \Delta {\text{log}}K^{\circ} = {\text{ log}}K^{\circ}_{{11}0 \, ({\text{reaction I}})} - {\text{ log}}K^{\circ}_{{111 }({\text{reaction II}})} $$

19 $$  \Delta \Delta {\text{G}}^{\circ} = \Delta {\text{G}}^{\circ}_{({\text{reaction I}})} - \, \Delta {\text{G}}^{\circ}_{({\text{reaction II}})} $$

20 $$  \Delta \Delta {\text{H}}^{\circ} = \Delta {\text{H}}^{\circ}_{({\text{reaction I}})} - \, \Delta {\text{H}}^{\circ}_{({\text{reaction II}})} $$

21 $$  \Delta \Delta {\text{S}}^{\circ} = \Delta {\text{S}}^{\circ}_{({\text{reaction I}})} - \, \Delta {\text{S}}^{\circ}_{({\text{reaction II}})} $$

The standard and differences thermodynamic parameters for all potential complexation processes are shown in Tables 7 and 8.

**Table 7:** Standard thermodynamic parameters of M^Z+^-phenylglycine complexes

Metal ion	T (°C)	Reaction with L^−^ (Scheme I)			Reaction with HL (Scheme II)		
		− ΔG° (kJ/mole)	ΔH° (kJ/mole)	ΔS° (J/deg. mole)	ΔG° (kJ/mole)	ΔH° (kJ/mole)	ΔS° (J/deg. mole)
UO_2_^2+^	25	40.51		344.26	11.70		110.54
	35	45.12	62.08	348.05	10.03	44.64	112.37
	45	47.37		344.18	9.50		110.50
La^3+^	25	22.31		504.53	31.61		360.37
	35	27.72	128.04	505.71	27.42	139.00	362.27
	45	32.39		504.50	24.42		360.32
Zr^4+^	25	58.48		77.58	− 6.16		− 145.44
	35	59.33	− 35.36	77.83	− 5.90	− 49.50	− 141.56
	45	60.04		77.61	− 3.23		− 145.50

Uncertainties are [ΔG°, 0.01 kJ mol^−1^; ΔH°, 0.01 kJ mol^−1^ and ΔS°, 0.02 J/deg. mole]

**Table 8:** The difference of thermodynamic parameters for M^Z+^-phenylglycine complexes

M^Z+^	T (°C)	Δ logK°	− ΔΔG° (kJ/mole)	ΔΔH° (kJ/mole)	ΔΔS° (J/deg. mole)
UO_2_^2+^	25	9.15	52.21		233.72
	35	9.35	55.15	17.44	235.68
	45	9.34	56.87		233.68
La^3+^	25	9.45	53.92		144.16
	35	9.35	55.14	− 10.96	143.44
	45	9.53	56.81		144.18
Zr^4+^	25	9.16	52.32		233.02
	35	9.06	53.43	14.14	219.39
	45	9.33	56.81		223.11

Uncertainties are [ΔG°, 0.01 kJ mol^−1^; ΔH°, 0.01 kJ mol^−1^ and ΔS°, 0.02 J/deg. mole]

The high negative ΔG° values for reaction of the metal ion with L^−^ (Scheme I) indicate that these complexation processes are spontaneous, while the complexation processes for reaction of the metal ion with HL (Scheme II) are nonspontaneous due to their positive ΔG° values [with exception of complexation of Zr^4+^ which characterized by its negative ΔH° values (i.e. have exothermic nature), therefore, it can be described as enthalpy favored processes] [9].

High negative ΔG° magnitudes for the reaction of Scheme I show that those complexation processes are spontaneous, whereas high positive ΔG° values for the reaction of Scheme II demonstrate that these complexation processes are nonspontaneous with the exception of Zr^4+^ complexation, which is distinguished by its negative ΔH° values (i.e., has an exothermic nature), therefore it can be described as an enthalpy-favored process [9]. The values of ΔlogK_1_°, ΔΔG°, ΔΔH° and ΔΔS° showed that the the reaction of the metal ion with L^−^ is the most prefer.

The high spontaneity of the metal ion's reaction with L-can be attributed to the electrostatic attraction across the two oppositely charged reactants, as shown by the following Eq. (6), which is supported by high ΔlogK_1_° values, even though L-reacting species typically have a lower concentration instead of HL reacting species (see Tables 2, 3, 4).

22 $$ {\text{M}}^{{\text{z}} +} + {\text{L}}^- \mathop{\longleftrightarrow} \limits^{K_{110} } {\text{ML}}^{{\text{z}} - 1} $$

When we looked at ΔS° data, we discovered that they were generally positive (with the exception of a few instances in which the metal ion reacts with HL, such as the complexation of Zr^4+^, which has negative ΔS° values). The large positive ΔΔS° values show that in all of the investigated cases, the standard entropy changes for the metal ion reaction with L^−^ were greater than those for the metal ion interaction with HL. This illustrates how the metal ion and L^−^ react more quickly. The strong negative ΔG° values for the reaction of the metal ion with L^−^ could be attributable to the greater value of the ΔS° term, which shows that these complexation processes are entropy favored processes. The positive “ΔΔS°” served to strengthen this conclusion. So, we will focus on the metal ion's reaction with L^−^ to identify a further component that regulates the complexation processes that are the subject of the investigation.

In the complexation processes, the nature of the metal ion is very important. By looking at ΔG° values, we can see that the complex structures' spontaneity and stability have increased in the following order: Zr^4+^ > UO_2_^2+^ > La^3+^.

We can infer that the stability of the formed complexes increased in the following order: Zr^4+^ > UO_2_^2+^ > La^3+^ by comparing the thermodynamic stability constants, Log K°_110_ (Scheme I) of M^Z+^-phenylglycine in the current work (Tables 5, 6) with that in our recent paper [9] as mentioned in Table 9. We can infer that the produced complexes' stability increased in the following order:

$$ {\text{Zr}}^{4 + } > {\text{Cu}}^{2 + } > {\text{UO}}_{{2}}^{{{2}+}} > {\text{Zn}}^{2 + } > {\text{Ni}}^{2 + } > {\text{ Cd}}^{2 + } > {\text{La}}^{3 + } $$

**Table 9:** Thermodynamic stability constants of M^2+^-phenylglycine complexes as presented in our previous paper [9]

Metal ion	log K°_110_ (Scheme I)			log K°_111_ (Scheme II)		
	25 °C	35 °C	45 °C	25 °C	25 °C	25 °C
Ni^2+^	5.08	5.06	5.00	− 4.31	− 4.40	− 5.00
Cu^2+^	9.22	8.96	8.62	− 0.24	− 1.38	− 1.12
Zn^2+^	5.44	5.66	5.10	− 4.02	− 3.90	− 4.23
Cd^2+^	4.44	5.03	3.94	− 5.03	− 4.31	− 5.39

With Irving–William's order [22], this is a nice agreement. Davies [23] asserts that an easy connection exists between the radius, r, of the unhydrated ion and its valence, z, as shown in Table 10, and the stability of the complexes that are generated. As a result, the large value of La3 + 's radius in compared to the other investigated metal ions is responsible for its less stable complexes than those of the other transition metals [24, 25]. The greater ratio of (valence/radius) and the Jahn–Teller effect lead to the conclusion that Zr^4+^ complexes are more reliable than those for other metal ions complexes [26–28].

**Table 10:** Ionic radii of studied metal ions

Metal ion	Radius (Ǻ)	z/r
Ni^2+^	0.72	2.78
Cu^2+^	0.69	2.90
Zn^2+^	0.74	2.70
Cd^2+^	0.97	2.06
Zr^4+^	0.86	4.65
UO_2_^2+^	0.66	3.03
La^3+^	1.06	2.83

## Conclusions

Using Bjerrum's potentiometric method, the interactions involving phenylglycine and a few transition metal ions (UO_2_^2+^, La^3+^ and Zr^4+^) in aqueous media were examined at three ionic strengths (μ = 0.05, 0.10 and 0.15 M KNO_3_) and three temperatures (25, 35, and 45 °C). The stability constants and thermodynamic functions have been calculated in order to identify the variables that govern the interaction between phenylglycine and the metal ions under investigation. In addition to other parameters such as the Jahn–Teller effect, it was discovered that these factors were related to the nature of the amino acid reactive species as well as to the nature of M^+^ such as the valence and radius of the ion. It was observed that reactions between the M^+^ and L^−^ were the most likely to occur. It was determined that the pH values affect the degree of complex formation ${\overline{\text{n}}}$ as well as the production of various reactive spices. When the range of degree of production was 0.5 < ${\overline{\text{n}}}$ < 1.15, forming 1:1 stoichiometric complex. Additionally, it was shown that the stability of the complexes produced between phenylglycine and MZ + increased in the subsequent order, which was in good accord with the Irving–Williams order:

$$ {\text{Zr}}^{{4} + } > {\text{Cu}}^{{2} + } > {\text{UO}}_{2}^{{2} + } > {\text{Zn}}^{{2} + } > {\text{Ni}}^{{2} + } > {\text{ Cd}}^{{2} + } > {\text{La}}^{{3} + } $$

With the exception of the enthalpy favored complexation processes of Zr^4+^, which are characterized by their negative ΔH° values, the complexation processes for reaction of the M^+^ with HL (Scheme II) are nonspontaneous due to their positive ΔG° values. The high negative ΔG° values for reaction of the metal ion with L^−^ (Scheme I) indicate that these complexation processes are spontaneous.

## Data Availability

The datasets used and/or analysed during the current study are available from the corresponding author on reasonable request.

## References

[1] Orabi AS, Abbas AM, Abd Elraoof MA, Khairy GM (2022). Modern view for binary and ternary complexes of metal ions with amoxicillin and some amino acids. Adv Environ Life Sci.

[2] Radalla AM (2022). Complexation equilibria and determination of stability constants of some divalent metal ion complexes of L-cysteine and diphenylamine in aqueous media. Am J Chem Eng.

[3] Liu X, Wu M, Li C, Yu P, Feng S, Li Y, Zhang Q (2022). Interaction structure and affinity of zwitterionic amino acids with important metal cations (Cd^2+^, Cu^2+^, Fe^3+^, Hg^2+^, Mn^2+^, Ni^2+^ and Zn^2+^) in aqueous solution. Molecules.

[4] Chetry N, Devi TG, Karlo T (2022). Synthesis and characterization of metal complex amino acid using spectroscopic methods and theoretical calculation. J Mol Struct.

[5] Gharakhloo M, Jagleniec D, Romanski J, Karbarz M (2022). A novel self-healing hydrogel based on derivatives of natural α-amino acids with potential applications as a strain sensor. J Mater Chem B.

[6] Yao W, Yang Z, Huang L, Su C (2022). Complexation of amino acids with cadmium and their application for cadmium-contaminated soil remediation. Appl Sci.

[7] Radalla AM, Mohamed A (2021). Potentiometric investigation on complex formation and stabilities of some divalent metal ions with L-cysteine and glycine as ligands in aqueous solutions. J Sol Chem.

[8] Rayah H, Absar B, Ghezzar MR, Abed F (2021). Complexation study of heavy metal ion Cu(II) with amino acids before elimination by ultrafiltration. Rasayan J Chem.

[9] Mohamed AA, El-Dossoki FI, Gumaa HA (2010). Thermodynamic study on the interaction between phenylglycine and some transition metal ions. J Chem Eng Data.

[10] Mohamed AA, Bakr MF, Abd El-Fattah HM (2003). Thermodynamic studies on the interaction between some amino acids with some rare earth metal ions in aqueous solutions. Thermochim Acta.

[11] Abd El-bary HM, Shata HA, Ez Elarab MAF, Mohamed AA, Emara MM (1996). Thermodynamics of amino acid ionization in aqueous solutions using pH-titration. J Indian Chem Soc.

[12] Bakr MF, Mohamed AA, Abd El-Fattah KA (2000). Thermodynamic studies on the complexation processes between some amino acids and some lanthanide metal ions in aqueous solutions. Al-Azhar Bull Sci.

[13] Bjerrum J (1957). *Metal amine formation in aqueous solutions*.

[14] Albert A, Serjeant EP (1971). The determination of ionization constants.

[15] Mohamed AA, Advanced An (2003). Thermodynamic study on the ionization processes of some amino acids. Al-Azhar Bull Sci.

[16] Christian DG (1980). Analytical chemistry.

[17] Meities L, Thomas LC (1958). Advanced analytical chemistry.

[18] Vogel AI (1978). Text book of quantitative inorganic analysis.

[19] Purcell KF, Kotz JC (1977). Inorganic chemistry.

[20] Irving H, Rossotti HS (1953). Methods for computing successive stability constants from experimental formation curves. J Chem Soc.

[21] Hartley FR, Burgess C, Alcock RM (1980). Solution equilibria.

[22] Barrett J (1993). Understanding inorganic chemistry.

[23] Davies CW (1962). Ion association.

[24] Cotton FA, Wilkinson G (1980). Advanced inorganic chemistry, a comprehensive text.

[25] Emara MM, Abd Elbary HM, Shehata HA, Ez El-Arab MAF, Mohamed AA (1995). L-serine interaction with some divalent and trivalent metals. Egypt J Appl Sci.

[26] Zhou J, Li R (2022). Introducing a new d^0^ Sc^3+^ asymmetric ion for functional materials: large birefringence enhancement by ScO_6_ in Ba_3_Sc_2_(BO_3_)_4_. Chem Phys Chem.

[27] Reddy KV (2002). Symmetry and spectroscopy of molecules.

[28] Grigoryan RA, Grigoryan LA, Babayan GG (2001). Synthesis of ZnFe_2_O_4_–Zn_2_ZrO_4_ solid solutions, inorganic materials.

